# Predictive model for prolonged length of hospital stay in patients with osteoporotic femoral neck fracture: A 5-year retrospective study

**DOI:** 10.3389/fmed.2022.1106312

**Published:** 2023-01-11

**Authors:** Worapaka Manosroi, Lattapol Koetsuk, Phichayut Phinyo, Pojsakorn Danpanichkul, Pichitchai Atthakomol

**Affiliations:** ^1^Division of Endocrinology, Department of Internal Medicine, Faculty of Medicine, Chiang Mai University, Chiang Mai, Thailand; ^2^Faculty of Medicine, Center for Clinical Epidemiology and Clinical Statistics, Chiang Mai University, Chiang Mai, Thailand; ^3^Department of Orthopaedics, Faculty of Medicine, Chiang Mai University, Chiang Mai, Thailand; ^4^Department of Family Medicine, Faculty of Medicine, Chiang Mai University, Chiang Mai, Thailand; ^5^Musculoskeletal Science and Translational Research Center, Chiang Mai University, Chiang Mai, Thailand; ^6^Department of Microbiology, Faculty of Medicine, Chiang Mai University, Chiang Mai, Thailand

**Keywords:** osteoporosis, femoral neck fracture, predictive model, length of hospital stay (LOS), prolonged

## Abstract

Prolonged length of stay (LOS) in osteoporotic femoral neck fracture patients increased the hospital care cost and demonstrated in-hospital complications. This study aimed to develop an ease-of use predictive model of prolonged LOS in osteoporotic femoral neck fracture patients. In this 5-year retrospective study, the medical charts of 255 patients admitted to hospital with an osteoporotic femoral neck fracture resulting from a simple fall from January 2014 to December 2018 were reviewed. Multivariable fractional polynomials (MFP) algorithms was applied to develop the predictive model from candidate predictors of prolonged LOS. The discrimination performance of predictive model was evaluated using the receiver operating characteristic curve (ROC). Internal validity was assessed using bootstrapping. From 289 patients who were hospitalized with an osteoporotic fracture of femoral neck throughout this study, 255 (88%) fulfilled the inclusion criteria. There was 54.90% (140 of 255 patients) of patients who had prolonged LOS. The predictors of the predictive model were age, BMI, ASA score class 3 or 4, arthroplasty and time from injury to surgery. The area under ROC curve of the model was 0.83 (95% confidence interval 0.77–0.88). Internal validation with bootstrap re-sampling revealed an optimism of −0.002 (range −0.300–0.296) with an estimated shrinkage factor of 0.907 for the predictive model. The current predictive model developed from preoperative predictors which had a good discriminative ability to differentiate between length of hospitalization less than 14 days and prolonged LOS in osteoporotic femoral neck patients. This model can be applied as ease-of use calculator application to help patients, their families and clinicians make appropriate decisions in terms of treatment planning, postoperative care program, and cost-effectiveness before patients receiving the definitive treatments.

## Introduction

Osteoporotic femoral neck fractures constitute approximately half of all osteoporotic hip fractures and are the type of fracture which has the highest demonstrated morbidity and mortality among all fractures (1–3). Osteoporotic femoral neck fracture are primarily caused by low energy trauma in the elderly, while in young adults the cause is typically the result of high-energy trauma such as motor vehicle accidents (4, 5). This type of injury usually requires surgery to restore normal function. In 1993 it was reported that the mortality rate of femoral neck fracture in elderly patients during the first year after surgery was approximately 30% (6). The causes of death were related to co-morbidities and postoperative complications (6). More recent data has indicated that the mortality rate of this type of fracture has declined to around 10% (7). One of the factors associated with the high mortality rates with this type of fracture is the length of stay (LOS). It has been reported that a hospital stays of more than 14 days (prolonged LOS) increases 30-day mortality in hip fracture patients (8).

Length of hospital stay is one of the important indicators in hip fracture care which are related to quality of treatment and which enable benchmarking (9). The burden of prolonged LOS in trauma patients is associated with in-hospital complications including urinary tract infection, pneumonia, sepsis, decubitus ulcer, delirium, and deep vein thrombosis (10). Increased hospital care cost has also been demonstrated in cases of prolonged LOS which had in-hospital complications (11). Therefore, earlier recognition of patients who are likely to have prolonged LOS can assist in the provision of effective and timely intervention. Earlier recognition can also help reduce the cost of hospitalization, the demand on medical resources and the incidence of patient in-hospital complications.

Various predictors for LOS in osteoporotic hip fractures have been reported including delay in time to surgery, previous hip fractures, cerebrovascular disease, smoking status, ASA classification, age and degree of decline in cognitive function (12–17). Some models to predict LOS in osteoporotic hip fracture patients have been proposed (18–20). One study created a model to predict LOS in hip fractures (both intertrochanteric and femoral neck types) (19). Another created a predictive model using multiple linear regression model but only in patients who underwent hip-replacement surgery (20). Another study developed a predictive model using machine learning algorithms in osteoporotic femoral neck fractures (18). However, although these proposed models are precise, they cannot be properly applied in clinical practice as the models use intra-and postoperative predictors to predict LOS which increases the time necessary to predict LOS (18). A practical predictive model should be able to be applied at an earlier time point to facilitate decisions by patients and their families as well as medical team preparations. A practical predictive model must be able to use preoperative predictors in model development.

Presently, there is no practical predictive model for prolonged LOS specifically for osteoporotic femoral neck fracture. This study aimed to develop an-easy-to-use model from preoperative predictors to predict prolonged LOS specifically for osteoporotic femoral neck fracture patients.

## Materials and methods

### Study design

This prognostic prediction research was conducted using a retrospective observational cohort design at an academic university hospital. The institutional ethics committee approved the study protocol (ethical number: ORT-2564-08118). The reporting of this study is compliant with the Transparent Reporting of a multivariable prediction model for Individual Prognosis Or Diagnosis (TRIPOD) statement (21).

### Study patients

Data of the patient sample were extracted from the medical records of the hospital. Patients who had a fractured neck of femur (ICD-10 = S7200) and who were admitted to the hospital between January 2014 and December 2018 were identified using International Classification of Disease 10 (ICD-10) codes. Two of staff members and the chief orthopedic resident conducted the retrospective chart reviews manually. Any debatable data was discussed and resolved by three orthopedic staff members. All data collectors have trained how to evaluate and gather the data from hospital medical records under supervision of the orthopedic staff members. Patient inclusion criteria were Thai patients, minimum age 50 years, and an osteoporotic femoral neck fracture injury deriving from a simple fall. The exclusion criteria were a bilateral femoral neck fracture, a prior femoral neck fracture and fractures in more than one area. Any possible pathological fractures or fractures caused by a high-energy mechanism including but not limited to traffic accidents were excluded from the study. Patients who died before discharge were also excluded.

Data on general demographics, types of comorbidities, results of laboratory investigations and type of surgical treatment were manually assembled from retrospective chart reviews. In cases where data could not be extracted from medical charts, telephone contact with the patient or their family was made to obtain the information.

### Data collection

Length of hospital stay was measured in days and was defined as the difference between the discharge and admission date (20). Prolonged LOS was defined as patients with a hospital stay greater ≥14 days (8, 12, 14). Generally, the discharge criteria for osteoporotic femoral neck fracture patients after surgery in our institute were: First, the patient can perform weight-bearing as tolerated with gait aids in patients who have undergone arthroplasty or partial weight-bearing with gait aids in patients who have undergone internal fixation (multiple screw fixation/dynamic hip screw). For patients who had dependent mobility before the fracture, ambulation with a wheelchair after surgery was the goal. Additionally, the body temperature should be less than 37.5 degree Celsius with no purulent drainage, pain or tenderness, localized swelling, or redness at the surgical site.

### Candidate predictors

• General factors
- Age at admission
- Gender
- Body mass index (BMI) at admission
- Independent mobility defined as a patient who could walk independently either with or without gait aids before the osteoporotic femoral neck fracture
• Types of comorbidities
- Active malignancy, dementia or Alzheimer’s disease, cerebrovascular disease, cardiovascular disease, congestive heart failure, chronic obstructive pulmonary disorder, diabetes mellitus, and current pneumonia
- American Society of Anesthesiologists (ASA) Physical Status Classification (ASA score) which were interpreted by anesthesiologists

The types of comorbidities in this study were determined based on the ICD-10 coding in the discharge summary.

• Laboratory investigation results
- Admission hemoglobin concentration
- Admission glomerular filtration rate (GFR)
• Characteristics of surgery
- Internal fixation, arthroplasty
- Time from injury to operation defined as the number of days from the date of the injury to the date of the operation.

### Statistical analysis

Statistical analysis was performed using STATA program (Stata/MP 16.1 for Mac, Copyright 1985–2019, Stata Corp., LLC, College Station, TX, USA). *P*-values < 0.05 were considered statistically significant. Categorical variables are presented as frequencies and percentages. Normally distributed continuous variables are presented as mean and standard deviation (SD). Non-normally distributed continuous variables are presented as median and interquartile range (IQR). Fisher’s exact test was used in calculating the associations between prolonged LOS and categorical variables. Student’s *t*-test was used in calculating the associations with normally distributed continuous variables. The relationship between non-normally distributed variables was analyzed using the Mann–Whitney *U*-test.

The study sample size was determined based on the standard recommendation of 10 events of interest per predictor variable (22). Thus, 140 prolonged length of stay status osteoporotic femoral neck fracture patients were needed for modeling 14 predictors. All continuous clinical predictors were retained to maintain completeness of data during the categorization of the continuous variables.

To develop the predictive model, multivariable fractional polynomials (MFP) algorithms were applied to avoid unnecessary categorization or inappropriate modeling of the determinants–outcomes association that would violate the linearity assumption (23). The MFP function allows investigators to identify the most appropriate functional form for each included continuous predictor. Log cumulative odd ratios (beta-coefficient), 95% confidence intervals (CI), and *P*-values are reported. Multiple imputation was applied if a candidate predictor had missing data >5% (24). The collinearity of each candidate predictor was assessed. Predictors which had a variant inflation factor (VIF) value >5 were excluded from the multivariable analysis. MFP consisted of two steps. The first step of MFP was employing the initial model approach by simultaneously including all candidate predictors within the model. Then, backward elimination was done to remove non-significant (*P*-value > 0.05) and non-contributing factors from the model, yielding the final model. In our study, however, we decided to preserve some candidate predictors based on prior clinical knowledge and previous published findings. In the second step, we determined the fittest fractional transformation for continuous covariates to be included during statistical modeling using a closed test algorithm.

To evaluate the model performance and for internal validation, the receiver operating characteristic (ROC) curve was plotted to assess the performance of the model. Hosmer–Lemeshow goodness-of-fit statistical testing was performed for model calibration. The calibration plot is presented to show the agreement between the actual observed event occurrence and the decile of the model-predicted probabilities. Internal validation was performed with a bootstrap re-sampling procedure with 1,000 replicates. The model optimism and shrinkage factor were estimated. The predictive model was developed into a calculator application. The application shows the predicted odds ratio and probability of prolonged LOS following osteoporotic femoral neck fracture after the input of all parameters.

## Results

Of the 289 patients hospitalized with an osteoporotic femoral neck during the time frame of this study, 255 (88%) met the inclusion criteria. Of that total, 54.90% (140 of 255) of the osteoporotic femoral neck fracture patients had prolonged LOS. The majority, 181 (70.98%), were female. The mean age was 75.65 ± 10.23 years. Arthroplasty was performed in most patients (216 or 84.71%). The median length of stay was 14 days. Demographic data are shown in Table 1.

**TABLE 1:** Baseline clinical characteristics and candidate predictors for prolonged length of stay of patients with osteoporotic femoral neck fracture (*n* = 255).

Characteristics	Missing data *n* (%)	Length of stay *n* (%)		*P*-value
		<14 days (*n* = 115)	≥14 days (*n* = 140)	
**General factors**				
Gender, *n* (%)	0 (0)			0.511
Male		31 (26.96)	43 (30.71)	
Female		84 (73.04)	97 (69.29)	
Age (years), Mean ± SD	0 (0)	73.83 ± 11.14	77.14 ± 9.20	0.011
BMI at admission (kg/meter^2^), Mean ± SD	1 (0.4)	20.92 ± 4.17	21.60 ± 3.52	0.162
Pre-fracture mobility, *n* (%)	55 (21.6)			0.293
Dependent mobility		3 (3.23)	7 (6.54)	
Independent mobility		90 (96.77)	100 (93.46)	
**Comorbidities**				
Active malignancy, *n* (%)	0 (0)	0 (0)	5 (3.57)	–
Dementia or Alzheimer’s disease, *n* (%)	0 (0)	4 (3.48)	13 (9.29)	0.075
Cerebrovascular disease, *n* (%)	0 (0)	11 (9.57)	18 (12.86)	0.412
Cardiovascular disease, *n* (%)	0 (0)	73 (63.48)	102 (72.86)	0.109
Congestive heart failure, *n* (%)	0 (0)	0 (1.87)	1 (0.71)	–
Chronic obstructive pulmonary disorder, *n* (%)	0 (0)	5 (4.35)	11 (7.86)	0.257
Diabetes mellitus, *n* (%)	0 (0)	30 (26.09)	41 (29.29)	0.571
Current pneumonia, *n* (%)	0 (0)	0 (0)	3 (2.14)	–
ASA score, *n* (%)	0 (0)			0.001
Class 1 or 2		88 (76.52)	78 (55.71)	
Class 3 or 4		27 (23.48)	62 (44.29)	
**Investigation factors**				
Hemoglobin (g/dl.), Mean ± SD	0 (0)	11.74 ± 1.72	11.15 ± 1.75	0.009
eGFR mL/min/1.73 m^2^, Mean ± SD	0 (0)	73.35 ± 29.98	60.36 ± 34.74	0.002
Type of surgery, *n* (%)	0 (0)			0.000
Internal fixation		30 (26.09)	9 (6.43)	
Arthroplasty		85 (73.91)	131 (93.57)	
Time from injury to surgery (days), Median (IQR)	0 (0)	5 (3–8)	13 (9–19.5)	0.000

BMI, Body mass index; ASA score, American Society of Anesthesiologists (ASA) Physical Status Classification; SD, standard deviation; IQR, interquartile range; eGFR = estimated glomerular filtration rate.

All candidate predictors exhibited a variant inflation factor (VIF) value <5. Multiple imputation was applied to the “dependent mobility” predictor. Multivariable fractional polynomial logistic regression was applied to all candidate predictors at the initial model. The area under the ROC curve of the initial model was 0.84. After back elimination, the final model predictors were age, BMI, ASA score class 3 or 4, arthroplasty and time from injury to surgery. The area under ROC curve of the final model was 0.83 (95% CI 0.77–0.88) (Table 2).

**TABLE 2:** Multivariable fractional polynomials logistic regression model of the initial model and the final model for prolonged length of stay in patients with osteoporotic femoral neck fracture.

Predictors	Initial model			Final model		
	Beta	95% CI	*P*-value	Beta	95% CI	*P*-value
**General factors**						
Male	−0.048	−0.743, 0.646	0.891		Not included	
Age	0.039	0.002, 0.076	0.039	0.042	0.008, 0.076	0.015
BMI	0.115	0.023, 0.207	0.014	0.104	0.019, 0.188	0.016
Dependent mobility	0.791	2.334, 0.751	0.315		Not included	
**Comorbidities**						
Active malignancy	–	–	–		Not included	
Dementia or Alzheimer’s disease	1.048	−0.236, 2.331	0.110		Not included	
Cerebrovascular disease	0.109	−0.968, 1.187	0.842		Not included	
Cardiovascular disease	−0.281	−1.018, 0.456	0.455		Not included	
Congestive heart failure	–	–	–		Not included	
Chronic obstructive pulmonary disorder	0.380	−0.938, 1.699	0.572		Not included	
Diabetes mellitus	0.339	−0.397, 1.075	0.366		Not included	
Current pneumonia	–	–	–		Not included	
ASA score class 3 or 4	0.558	−0.170 1.287	0.133	0.641	−0.007, 1.288	0.052
**Investigation factors**						
Hemoglobin concentration	−0.052	−0.259, 0.154	0.621		Not included	
eGFR	−0.004	−0.015, 0.007	0.459		Not included	
**Type of treatment**						
Internal fixation		Reference			Reference	
Arthroplasty	1.270	0.265, 2.275	0.013	1.247	0.305, 2.188	0.009
Time from injury to surgery	10.104	6.758, 13.450	0.000	10.217	6.955, 13.479	0.000
Model Intercept (constant)	0.086	−1.693, 1.865	0.925	−0.673	−1.608, 0.261	0.158

Area under ROC curve of the initial model = 0.84. Area under ROC curve of the final model = 0.83. BMI, Body mass index; ASA score, American Society of Anesthesiologists (ASA) Physical Status Classification; eGFR, estimated glomerular filtration rate.

**FIGURE 1:**
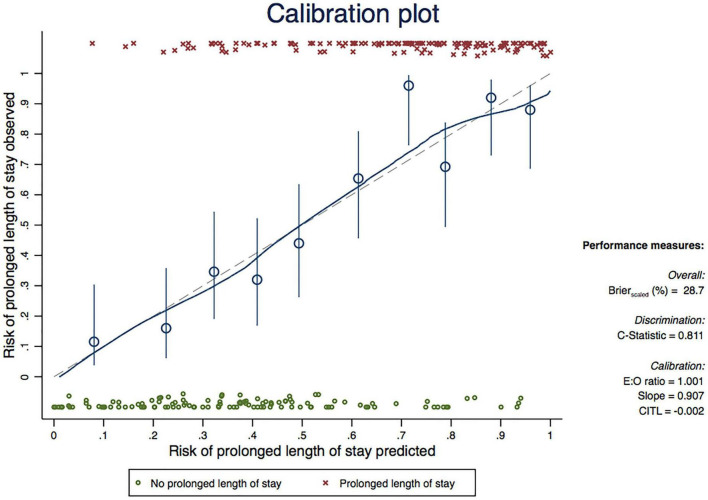
Calibration plot comparing observed and predicted risk of prolonged length of stay in patients with osteoporotic femoral neck fracture (based on final model).

The fittest fractional transformation for continuous variables from the final model is shown in Table 3. The calibration of the final model is presented with calibration plots (Figure 1). The Hosmer–Lemeshow goodness-of-fit statistic was insignificant for the final model (*p* = 0.491). Internal validation with bootstrap re-sampling revealed an optimism of −0.002 (range −0.300–0.296) with an estimated shrinkage factor of 0.907 for the final model.

**TABLE 3:** Multivariable fractional polynomial logistic regression model for prolonged length of stay in patients with osteoporotic femoral neck fracture (final model).

Predictors	Covariate transformation			Beta	95% CI	*P*-value
	Term	df	Formula			
Intercept				−0.673	−1.608, 0.261	0.158
Age	Linear	1	Age-75.646	0.042	0.008, 0.076	0.015
BMI	Linear	1	BMI-21.290	0.104	0.019, 0.188	0.016
ASA score class 3 or 4	Linear	1		0.641	−0.007, 1.288	0.052
Arthroplasty	Linear	1		1.247	0.305, 2.188	0.009
Time from injury to surgery	FP2	2	0.5[(Time + 1)/ 100]−0.360	10.217	6.955, 13.479	0.000

Area under ROC curve of the final model = 0.83. BMI, Body mass index; ASA score, American Society of Anesthesiologists (ASA) Physical Status Classification.

**FIGURE 2:**
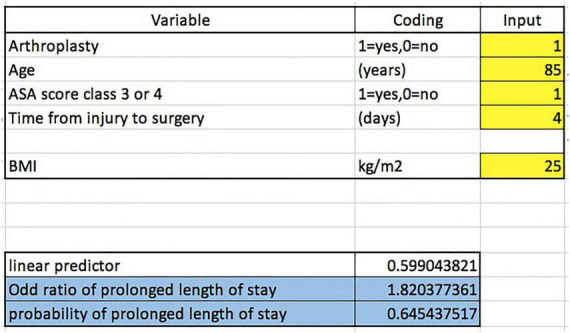
The predicted odds ratio and probability of prolonged LOS for an osteoporotic femoral neck fracture patient who will receive arthroplasty who is age 85, has an ASA score class three, is schedule for surgery 4 days after falling and has a BMI = 25 kg/m^2^.

The final model was developed into a calculator application of the predicted odds ratio and probability of prolonged LOS following osteoporotic femoral neck fracture (Supplementary Appendix 1). For example, the predicted odds ratio and probability of prolonged LOS following osteoporotic femoral neck fracture of a patient who will receive arthroplasty who is age 85, has ASA score class 3, is scheduled for surgery 4 days after falling and BMI = 25 kg/m2 are 1.820 and 0.645, respectively (Figure 2).

## Discussion

Currently there are no models which can predict prolonged LOS in osteoporotic femoral neck fracture patients during the preoperative period. Our results demonstrate that age, BMI, ASA score class 3 or 4, arthroplasty and time from injury to surgery are strong predictors of prolonged LOS. This predictive model could help patients, their families and clinicians in making the decisions related to treatment planning, postoperative care programs and maximizing cost-effectiveness. This model has shown good to excellent discriminative performance and was well calibrated during internal validation.

Many factors have been reported to be predictors of prolonged LOS in patients with hip fractures or who are receiving hip surgeries. Those factors, similar to our study, include age, time to surgery, and ASA score as factors influencing LOS (12–14, 16, 17, 25–30). Additionally, many types of comorbidities, e.g., such as anemia, urinary disorders, cardiovascular diseases, chronic obstructive pulmonary disease, chronic cognitive impairment/dementia, and acute renal failure (12, 16, 17, 20, 31) have been reported to be predictors related to prolonged LOS. We found that ASA score alone can be used to represent the overall comorbidity situation as it is a grading system for evaluating the preoperative health of surgical patients (32). One study reported a negative association between BMI and increased LOS (18). That result is in discordance with our study which showed high BMI is related to increased LOS. There are, however, several hypotheses supporting our results. First, higher BMI is associated with metabolic syndrome, indicating an increased risk of several postoperative complications in geriatric hip fracture patients (33, 34). Second, high BMI can make the surgical approach more difficult (35) which could disturb soft tissue vascularity leading to increased time for postoperative wound care. There is evidence that patients with hip arthroplasty have 3.3 days longer hospitalization compared to patients with osteosynthesis (27) which supports our finding that performing arthroplasty increases the risk of prolonged LOS. As to the choice of implants in a femoral neck fracture, internal fixation is generally recommended in cases of non-displaced fracture as there is a lower chance of disrupting the femoral head blood supply. In displaced femoral neck fractures, arthroplasty is recommended for older individuals with displaced fractures (36). Our results show time to surgery to be the only modifiable predictor associated with prolonged LOS in patients with femoral neck fracture.

Our predictive model had good to excellent ability to differentiate osteoporotic femoral neck patients who would have prolonged LOS during their admission from patients who would have LOS <14 days with the area under the ROC curve of 0.83 (95% CI 0.77–0.88). That good to excellent discriminative performance might be explained by the appropriate fractional polynomial modeling of the predictors. Our model was also well calibrated during internal validation (optimism of −0.002, range −0.300–0.296). Compared to other predictive models, our model has a higher accuracy than the multiple linear regression model from Trunfio et al. which achieved an overall accuracy of between 0.651 and 0.718 (20) and is comparable to the model by Knoll et al. which had an accuracy of within 3 days of the true LOS for 0.758 of their series (19). There are numerous variations among predictive models in terms of inclusion criteria, candidate predictors and type of regression model that could potentially affect the accuracy of each model (19, 20).

Strengths of this study include the following. First, this model is specific to osteoporotic femoral hip fracture patients without the limitation to specific treatments as in previous models (20). Thus, patients who plan to receive multiple screw fixation can use our model to predict the probability of prolonged LOS (19). Second, the point of prediction of the calculator application based on this model can predict probability of prolonged LOS at the day of admission. The predictors, including age, BMI and ASA score of patients, can be simply included in the calculator application. Whether to perform multiple screw fixation or arthroplasty is dependent on the patient’s age and fracture configuration (36), while time from injury to surgery is dependent on the preoperative patient’s condition and the schedule of the operating theater. Using this new model, patients and their families can make decisions regarding treatment after discussion with the medical team. In cases where a prolonged length of stay is predicted and they cannot afford the cost of hospitalization, they can seek other less expensive medical institution options.

There are some limitations in this study. First, some intra- or postoperative predictors reported in previous studies (12, 18), e.g., excessive intravenous glucose and sodium chloride infusion after surgery, excessive intraoperative bleeding, postoperative acute renal failure, ventilator use >48 hours, postoperative pneumonia, and other postoperative complications, could potentially increase the discriminative performance of the model. However, we considered that the benefit from prediction of prolonged LOS at the date of admission to be more valuable than prediction of prolonged LOS made during the postoperative period. For that reason, we did not include these predictors in our model. Second, although our predictive model has good to excellent discriminative performance and well-calibrated internal validation, for generalizability, temporal and external validation of the predictive model should be assessed. Third, we could not include all types of comorbidities as candidate predictors due to the limitation of sample size.

## Conclusion

The current predictive model, developed from preoperative predictors including age, BMI, ASA score class 3 or 4, arthroplasty and time from injury to surgery, had a good ability to differentiate between length of hospitalization less than 14 days and prolonged LOS in osteoporotic femoral neck patients. Clinical implementation of this model can be accomplished as an ease-of use calculator application which can help patients, their families and clinicians make appropriate decisions regarding treatment, postoperative care and cost-effectiveness before deciding on a definitive course of treatment.

## Data availability statement

The raw data supporting the conclusions of this article will be made available by the authors, without undue reservation.

## Ethics statement

The studies involving human participants were reviewed and approved by the Faculty of Medicine, Chiang Mai University. Written informed consent for participation was not required for this study in accordance with the national legislation and the institutional requirements.

## Author contributions

PA and WM initiated conception and design of the study, performed the collection and acquisition of data, performed the data analysis with interpretation, wrote the manuscript, and were responsible for critical revision. LK was involved in the process of data collection, acquisition, interpretation, and manuscript editing. PP performed the data analysis, interpreted the data, and edited the manuscript. PD participated in data interpretation and manuscript editing. All authors contributed to the article and approved the submitted version.

## Supplementary material

Supplementary Appendix 1 Calculator application of the predicted odds ratio and probability of prolonged LOS following osteoporotic femoral neck fracture.
